# The Mysteries of Primary Amenorrhea: Swyer Syndrome

**DOI:** 10.7759/cureus.28170

**Published:** 2022-08-19

**Authors:** Srinidhi Cherukuri, Shubhada S Jajoo, Deepika Dewani, Manisha Andela

**Affiliations:** 1 Obstetrics and Gynaecology, Jawaharlal Nehru Medical College, Datta Meghe Institute of Medical Sciences, Wardha, IND

**Keywords:** gonadectomy, gonadoblastoma, gonadal dysgenesis, primary amenorrhea, swyer syndrome

## Abstract

Swyer syndrome is a hereditary condition seen in a few patients who present with primary amenorrhea, characterized by 46 XY and the presence of female internal genital tract and bilateral streak gonads in a female phenotype. A 25-year-old lady presented with primary amenorrhea. After the evaluation, she was diagnosed with Swyer syndrome due to a mutation in the SRY gene, leading to failure of testicular development. The clinical presentation was that of a female phenotype with no secondary sexual characteristics. On physical examination, she had a female phenotype with a short vagina and no secondary sexual characteristics. The MRI revealed a hypoplastic uterus with both fallopian tubes but with streak gonads. The patient's genotype was found to be 46 XY after genetic testing. The streak gonads were removed laparoscopically due to the future risk of gonadoblastoma, and the patient was given hormone replacement therapy (HRT). The patient started menstruating after six months of HRT and has been developing secondary sexual characteristics (Tanner stage II) till now.

## Introduction

Swyer's disease, also called pure gonadal dysgenesis, was later related to dysgenetic gonads. A deletion in the SRY gene's DNA-binding region 2 is present in 10 to 20 percent of women with Swyer syndrome. However, the SRY gene is normal in 80-90 percent of cases, and mutations in other testis-determining variables are also likely to be involved. These patients are phenotypically feminine, with phenotypically female genital morphology at birth and normal sexual Mullerian structure [1]. Due to the lack of hormonal or reproductive capacity, the disorder commonly manifests in adolescence with delayed puberty and amenorrhoea. Despite the high occurrence of gonadoblastoma and germ cell cancers, the current standard of care is to undergo gonadal removal without delay after diagnosis [1].

This syndrome has a high propensity for tumor formation, with an incidence of 20-30%. The most common tumor is gonadoblastoma, which is frequently bilateral, but dysgerminoma and even embryonal cancer are also found [2]. About 5% are phenotypic females with defective gonads and the 46 XY karyotype [2].

Swyer syndrome is treated similarly to other types of ovarian dysfunction, with estrogen supplementation to produce secondary sexual characteristics and long-term combined estrogen and progesterone replacement therapy. Due to a lack of gonadal steroid production, Swyer syndrome patients have a severe type of estrogen insufficiency [1].

## Case presentation

**Figure 1:**
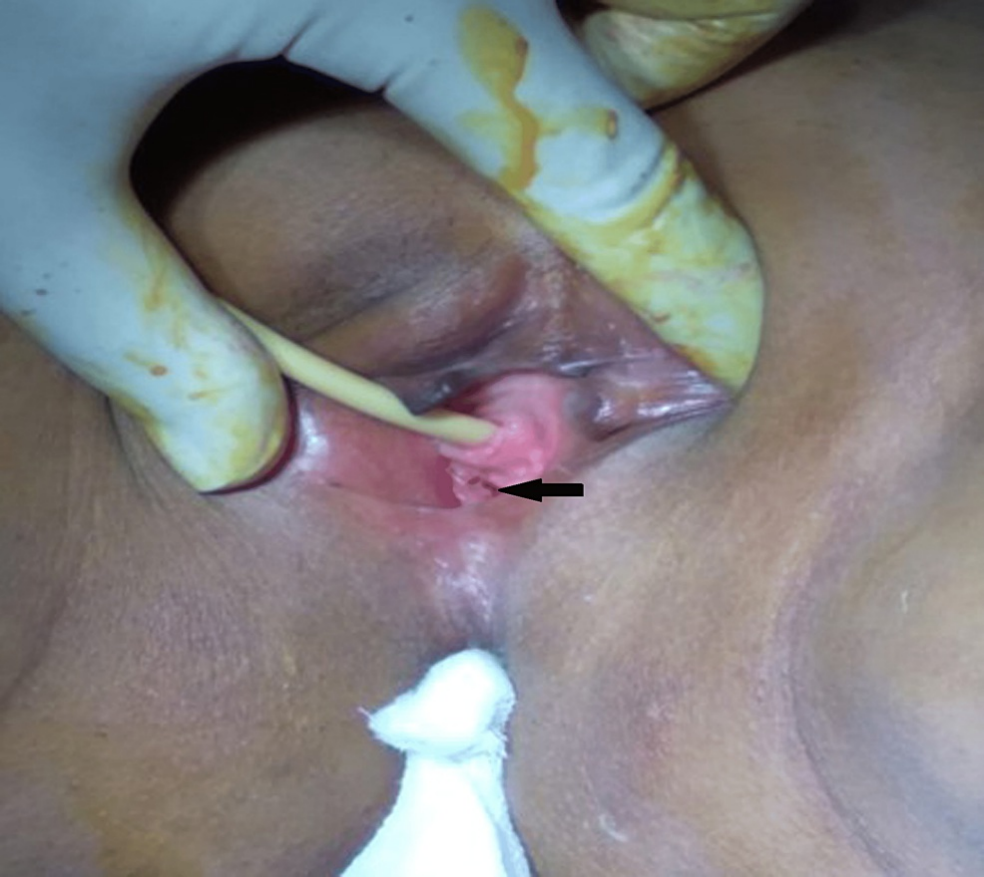
Local examination showing hymen opening.

A 25-year-old woman with primary amenorrhea presented to the gynecological outpatient specialization. A clinical profile showed a BMI of 18 kg/m^2^ with an absence of pubic and axillary hair and no breast development. The abdomen was soft with no palpable mass. The external genitalia is female and appeared normal with a hymenal opening (Figure 1).

**Figure 2:**
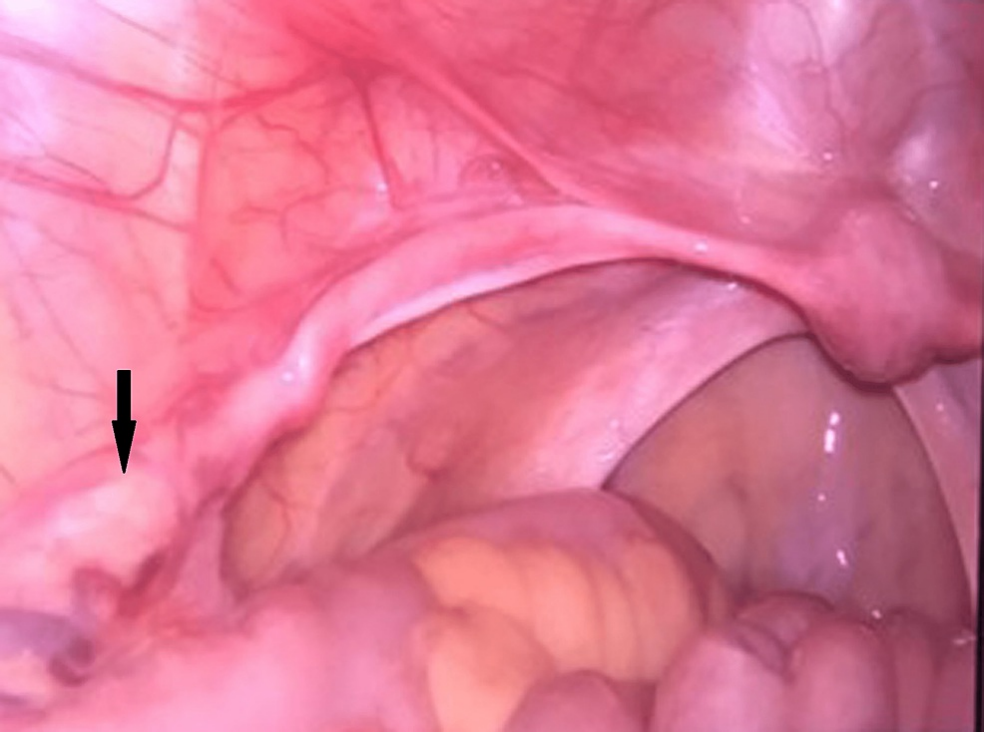
Laparoscopy image showing streak gonads (whitish fibrous bands).

**Figure 3:**
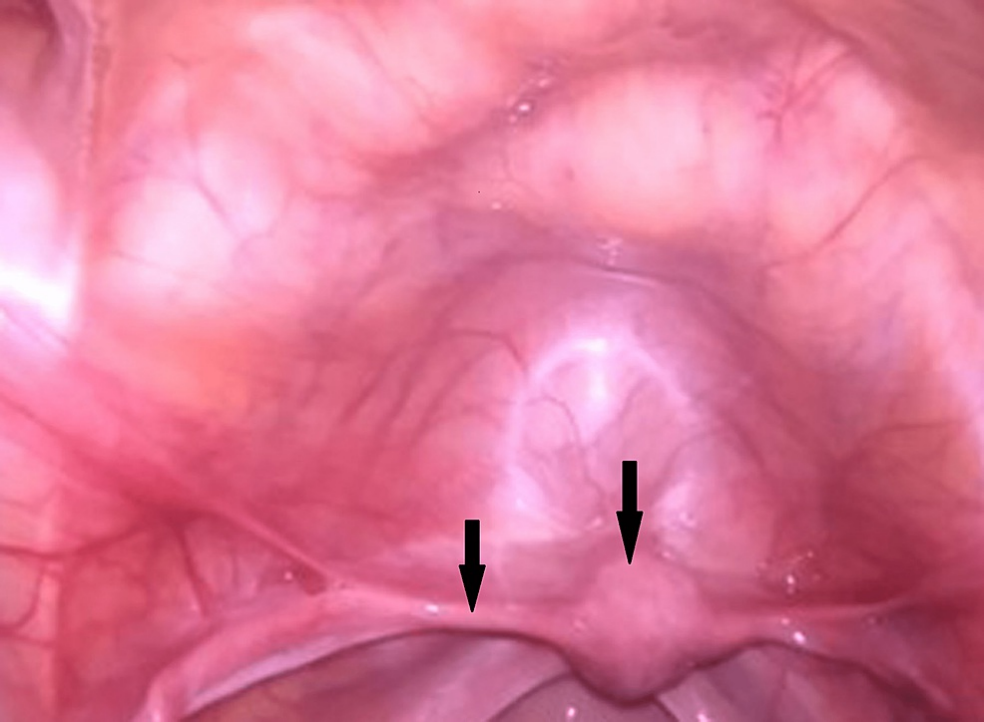
Laparoscopy image showing uterus, fallopian tube.

On investigations, her hormonal profile revealed serum follicle-stimulating hormone (FSH) - 50.37 ng/dl (puberty: 0.3-10 mIU/ml, adult: 1.5-12.4 mIU/ml), serum luteinizing hormone (LH) - 19.03 ng/dl (male: 1.2-8.6 mIU/ml, female: 1.2-103 mIU/ml), serum prolactin - 8.3 ng/dl (up to 25 mcg/l), and serum testosterone - 6 ng/dl (7-77 ng/dl). These tests were done to identify her ovarian reserves to rule out Mullerian agenesis and testicular feminization. Ultrasonography of abdomen and pelvis showed grossly hypoplastic thin rudimentary uterus with upper vagina canalized; both ovaries were not visualized. The MRI pelvis showed a hypoplastic uterus of approximately 15 x 23 x 34 mm (APxTRxCC), the endometrial thickness of about 4.5 mm, with no visualization of bilateral gonads. The patient was subjected to chromosomal analysis, which revealed 46 XY karyotype. After giving informed consent, the patient was taken for diagnostic laparoscopy and evaluation under general anesthesia. An infantile uterus, fallopian tubes (Figure 2), and streak gonads (Figure 3) that appeared like whitish fibrous bands were visualized but did not visualize ovaries. The inguinal canal was visible on both sides; however, it was empty. No other pelvic structural abnormalities were noted.

**Figure 4:**
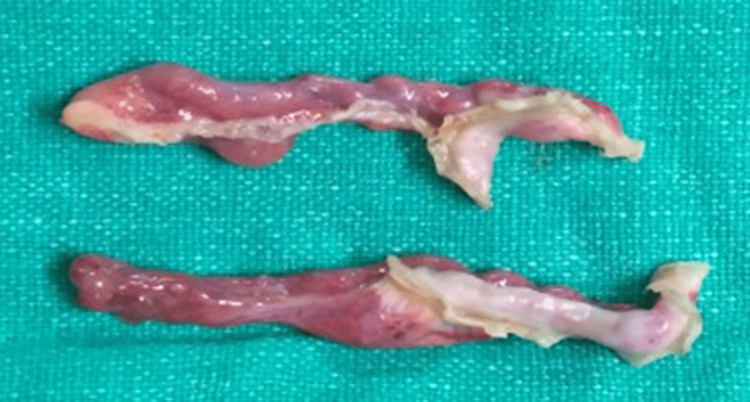
Specimen showing streak gonads.

The inguinal canal was visible on both sides; however, it was empty. No other pelvic structural abnormalities were noted. The karyotyping, radiological, and intraoperative findings were informed and discussed with the patient's parents. Considering the obtained karyotype (46, XY) of the patient with the above laparoscopic findings, the white fibrous band-like (streak gonad) structures (Figure 4) were removed bilaterally and sent for histopathological examination. The histopathological report showed ovarian differentiation and dysgenetic streak gonads.

Postoperatively, the patient was counseled and started on hormone replacement therapy for the first 90 days. She was first given conjugated estrogen and then started on a combination of conjugated estrogen and medroxyprogesterone acetate on a cyclical basis. The patient started menstruating after six months of hormone replacement therapy (HRT) and has been developing secondary sexual characteristics (Tanner stage II) till now.

## Discussion

Swyer syndrome is a type of gonadal dysgenesis that affects only the testes. The formation of testes is the first recognized step in a normal XY fetus's sexual differentiation. Along with multiple genes, SRY on the Y chromosome is required during the initial phases of testicular formation in the 2nd month of pregnancy. Four mutations in the SRY gene cause Swyer syndrome. The cause is usually unknown, but it could be caused by mutations in other sex-differentiating genes on the X chromosome, including the autosomal genes. The bipotential gonads in an XY fetus fail to develop into testes when these genes are dysfunctional, which leads to non-virilization of external genitalia due to no production of testosterone or anti-Mullerian hormone (AMH), giving genitalia of typical female appearance. Because the AMH is absent, the Mullerian ducts develop into the uterus, fallopian tube, cervix, and vagina [3]. Primary amenorrhea is described as the absence of menstruation by 16 years of age if secondary sexual development is normal or by 14 years if secondary sexual development is not normal [4]. Swyer syndrome is an uncommon disease that causes primary amenorrhea in women. Swyer syndrome patients have a female genital morphology and are phenotypically female. In clinical terms, these women have a 46 XY karyotype, impaired sexual development, and primary amenorrhea.

Because of insufficient pubertal development, 46 XY gonadal dysgenesis is generally detected in early adolescence. The external genitalia, uterus, and fallopian tubes of a female can all be seen. Raised gonadotropin, estrogen at a decreased level, and androgen levels within the normal female range have all been found in laboratory tests [4]. Non-testicular feminization is evident with androgen insensitivity at the level of the target organ, with the karyotype 46 XY. In true hermaphroditism, gonads (ovotestis) with both testicular and ovarian components are seen. The differential diagnosis was true hermaphroditism because our patient lacked ovarian tissue; hypergonadotropic hypogonadism differentiated our case from testicular feminization [4]. The chance of developing gonadoblastoma is about 25 percent with this syndrome. Following confirmation of the diagnosis, our patient with Swyer syndrome underwent gonadectomy, and a histopathological examination was conducted. In our case, we began with 2 mg of estradiol to accelerate puberty and promote the growth of secondary sexual characteristics. She was then switched to estrogen and progesterone once secondary sexual differentiation and endometrial mucosal thickness had occurred.

## Conclusions

The MRI revealed a hypoplastic uterus with both fallopian tubes but with streak gonads. The patient's genotype was found to be 46 XY after genetic testing. The streak gonads were removed laparoscopically due to the future risk of gonadoblastoma, and the patient was given hormone replacement therapy. The patient started menstruating after six months of HRT and has been developing secondary sexual characteristics (Tanner stage II) till now.
